# ACCELERATED AGING IN MID LIFE: BEHAVIORAL, EMOTIONAL, AND PHYSIOLOGICAL MARKERS OF AGING

**DOI:** 10.1093/geroni/igad104.0912

**Published:** 2023-12-21

**Authors:** Maayan Agmon

## Abstract

The aging process starts at midlife and is multifactorial, involving behavioral, emotional, and biological pathways. Most studies to date have focused on either older ages, when age-related diseases already are present, or a single mechanism, thus missing the full scope of the aging process. The proposed symposium aims to fill this gap by highlighting the many-sided nature of aging among midlife adults, especially the contributions of behavioral, emotional, and biological markers. We will discuss how physical capacity, sensory modulation, and exposure to violence are associated with biological age, reflecting the true state of the body and the diversity of aging. Understanding these relationships can both illuminate early markers of the aging process and support the design of tailored interventions to reduce the prevalence of accelerated aging. The symposium will present the latest research on the combined markers to gain insight into normal and accelerated aging. Professor Agmon will give an overview of newly developed behavioral markers that quantify the aging process, such as the photogrammetry-based analysis of posture and physical capacity battery. Roy Tzemah-Shahar will discuss the relationships between physical capacity metrics and rate of aging, Merav Asher will explicate the association between one’s sensory profile and the rate of aging, and Khalil Iktilat will share how exposure to violence explains the accelerated aging of midlife Muslims. Finally, we will discuss the need for more research on midlife adults and the potential benefits of early interventions.

